# Overexpression of the *Arabidopsis* MACPF Protein AtMACP2 Promotes Pathogen Resistance by Activating SA Signaling

**DOI:** 10.3390/ijms23158784

**Published:** 2022-08-07

**Authors:** Xue Zhang, Yang-Shuo Dai, Yu-Xin Wang, Ze-Zhuo Su, Lu-Jun Yu, Zhen-Fei Zhang, Shi Xiao, Qin-Fang Chen

**Affiliations:** 1 State Key Laboratory of Biocontrol, Guangdong Provincial Key Laboratory of Plant Resources, School of Life Sciences, Sun Yat-sen University, Guangzhou 510275, China; 2 Plant Protection Research Institute Guangdong Academy of Agricultural Sciences, Guangzhou 510640, China; 3 School of Biomedical Sciences, Li Ka Shing Faculty of Medicine, The University of Hong Kong, Pokfulam, Hong Kong SAR, China; 4 Laboratory of Data Discovery for Health Limited (D24H), Hong Kong Science Park, Hong Kong SAR, China

**Keywords:** MACP2, membrane attack complex/perforin-like protein, pathogen infection, salicylic acid signaling, indole glucosinolates

## Abstract

Immune response in plants is tightly regulated by the coordination of the cell surface and intracellular receptors. In animals, the membrane attack complex/perforin-like (MACPF) protein superfamily creates oligomeric pore structures on the cell surface during pathogen infection. However, the function and molecular mechanism of MACPF proteins in plant pathogen responses remain largely unclear. In this study, we identified an Arabidopsis MACP2 and investigated the responsiveness of this protein during both bacterial and fungal pathogens. We suggest that MACP2 induces programmed cell death, bacterial pathogen resistance, and necrotrophic fungal pathogen sensitivity by activating the biosynthesis of tryptophan-derived indole glucosinolates and the salicylic acid signaling pathway dependent on the activity of enhanced disease susceptibility 1 (EDS1). Moreover, the response of MACP2 mRNA isoforms upon pathogen attack is differentially regulated by a posttranscriptional mechanism: alternative splicing. In comparison to previously reported MACPFs in Arabidopsis, MACP2 shares a redundant but nonoverlapping role in plant immunity. Thus, our findings provide novel insights and genetic tools for the MACPF family in maintaining SA accumulation in response to pathogens in Arabidopsis.

## 1. Introduction

As sessile organisms, plants have evolved sophisticated mechanisms to communicate with surrounding microorganisms, including beneficial and pathogenic interaction. Plant pathogens secrete effector proteins to suppress host immune responses during their colonization [1,2]. Thus, plant cells are equipped with a variety of cell surface or intracellularly localized receptor proteins that can recognize microorganisms and initiate downstream immune responses to restrict pathogen proliferation [3]. In particular, cell surface receptors, also known as pattern recognition receptors (PRRs), function in the recognition of conserved pathogen-associated molecular patterns (PAMPs) and are able to trigger PAMP-triggered immunity (PTI) responses during plant interactions with either nonpathogenic or pathogenic microbes [3,4]. For example, PAMP receptors such as PRR FLAGELLIN SENSING 2 (FLS2), chitin elicitor receptor kinase 1 (CERK1), and EF-Tu receptor (EFR) recognize conserved microbial effectors, flagellin (or the minimal epitope flg22), fungal chitin, and elongation factor thermo unstable (EF-Tu), respectively, in *Arabidopsis* [3,5]. Furthermore, PAMP receptors form heterocomplexes with coreceptors such as Brassinosteroid insensitive 1-associated kinase 1 (BAK1) to activate downstream responses under microbial infection [3]. In addition to PTI, plants exhibit counter-defense strategies to initiate a locally rapid immune response to microbial-derived effectors through intracellular nucleotide-binding domains and leucine-rich repeat-containing receptors (NLRs). Specifically, a typical NLR consists of a Toll/interleukin 1-receptor (TIR) or coiled-coil domain at the N-terminus, leucine-rich repeats (LRR) at the C-terminus, and an internal nucleotide-binding region [6,7]. The NLR-mediated strategy is characterized as effector-triggered immunity (ETI) and is frequently linked with the hypersensitive response (HR) [8], a form of programmed cell death (PCD) [9]. To date, HR has been considered an efficient and immediate immune reaction in response to pathogen invasion, leading to rapid cell death to limit pathogen proliferation at the entry site [10]. However, little is known about the underlying mechanism of this response in plant immunity. Recent studies have suggested that the plant defense hormone salicylic acid (SA) emerges as a pivotal signal to mediate immunity-related HR, linking the activation of pathogenesis-related (PR) genes and HR-induced PCD to confer resistance to pathogens [11,12,13]. In particular, plant mutants with misregulation of the cell death pathway result in lesion-mimicking phenotypes and the constant activation of SA signaling, H_2_O_2_ accumulation, and PR genes [7,14,15]; these materials are powerful tools for studying the underlying connection between PCD and plant immunity [15].

While the HR can be a rapid response for containing disease progress at the site of pathogen entry, mechanisms that cause microbial death remain to be investigated. In recent years, mammalian pore-forming proteins have been found to target and lyse infected microorganisms by forming the membrane attack complex (MAC), an oligomeric cylindrical ring at the surface of target membranes [16]. Since then, phylogenetic analysis has indicated that the conserved signature of the membrane attack complex and perforin (MACPF) proteins, i.e., Y/S-G-T/S-H-X7-G-G (X), is present in both eukaryotic and prokaryotic organisms to form the MACPF superfamily [17,18]. The members of this family have been documented to play essential roles in diverse developmental processes and immune responses [19]. MACPF proteins—which are related to cholesterol-dependent cytolysins and are structurally similar to C6, C7, C8α, C8β, and C9 complement system proteins [20]—can form cellular membrane pores and perform biological functions [21,22]. The formation of pore structures causes a breach of cellular integrity, ultimately inducing cell death by allowing the free passage of molecules in/out of the corresponding cell [19]. Several MACPF proteins have been structurally resolved with conserved MACPF oligomers and varied C-terminal domains [23].

However, there is little information related to the biological function of plant MACPF domain-containing proteins. In particular, several reports have indicated that MACPF proteins play important roles in viral and bacterial infections and plant PAMP-triggered immunity [1,4,23]. Many plant *MACPF* genes have been identified in *Arabidopsis* [15], *Poaceae* species [18], and cotton [24] and have been divided into four groups using phylogenetic analysis and domain organization [18]. Several MACPF genes (such as C6, C7, C8α, C8β, and C9) have been experimentally identified in animals with functions in growth and immunity [19,21,25,26,27]. The published transcriptomic data indicate that *MACPF* genes are involved in plant growth, development, and response to biotic and abiotic stresses [18]. In contrast, few genes have been experimentally confirmed in plants. For example, in *Gossypium*, silencing of the *GhMACPF26* gene enhanced tolerance of cotton plants to cold stress [24]. *Arabidopsis* constitutively activated cell death 1 (CAD1), localized to both the plasma membrane and cytosol, is a salicylic acid (SA)-responsive protein, which plays important roles in immunity-induced PCD [10,15,28] and could influence endophytic phyllosphere microbiota [29]. The *cad1* mutant shows a lesion phenotype that mimics HR and is also regulated by chitin elicitors independent of the SA-mediated pathway [10]. Similarly, another *Arabidopsis* membrane-localized necrotic spotted lesion 1 (NSL1) took part in SA-mediated defense responses and PCD. The *nsl1* mutant showed spotted necrotic lesions, retarded plant growth, and high accumulation of SA, thus activating the SA signaling pathway and linking PAMP-induced PCD to antimicrobial metabolism upon pathogen attack [4,30].

A total of four MACPF proteins have been identified in Arabidopsis; however, in addition to CAD1 and NSL1, the other two MACPFs have not been studied well. To this end, we isolated two independent mutants of *AtMACP2* (At4g24290) from *Arabidopsis*. Phenotypic and genetic analysis suggested that MACP2 is involved in the SA-mediated PCD response during pathogen infection and that the activation of SA signaling may result from altered metabolism of tryptophan (Trp)-derived indole glucosinolates. Further analysis indicated that MACP2 undergoes posttranscriptional regulation by alternative splicing (AS), and the three spliced isoforms detected based on the database information differentially respond to treatment with bacterial and fungal pathogens, implying distinct responsive pathways derived from plant immunity.

## 2. Results

### 2.1. Characterization of T-DNA Insertional Mutants and Transgenic Overexpression Lines in MACP2

**Figure 1:**
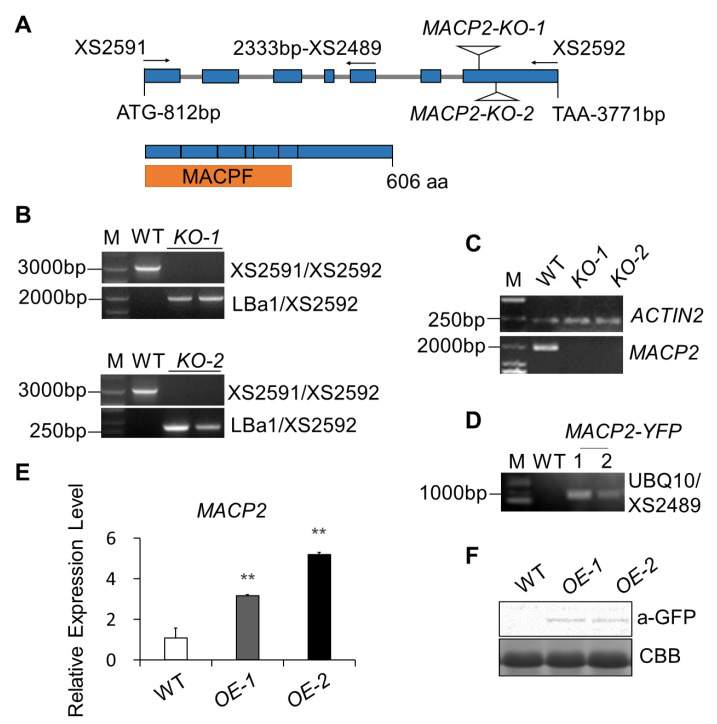
Characterization of T-DNA insertional mutants and transgenic overexpression lines in *MACP2.* (**A**) Schematic depicting the MACP2 gene, indicating the T-DNA insertion site of *MACP2-KO-1* and *MACP2-KO-2.* (**B**) Identification of *MACP2-KOs* via PCR. The full-length *MACP*2 was amplified with primer pair XS2591 and XS2592. The length-contained T-DNA sequence was amplified via primer pair LBa1 and XS2592 in *KO-1*, and *KO-2*. (**C**) Semiquantitative PCR of *MACP2* in WT and *MACP2-KOs*. The full-length *MACP2* was amplified with primer pair XS2591 and XS2592. The *ACTIN2* was amplified with primer pair ACTIN2-F and ACTIN2-R. (**D**) Identification in DNA level of *MACP2-YFP* transgenic plants. MACP2 CDS was cloned into pFGC-RCS binary vector then the expression cassette of MACP2-YFP was inserted into the Arabidopsis genome. UBQ10 and XS2489 were derived from the pFGC-RCS plasmid and MACP2 CDS, respectively. (**E**) Identification in RNA level of *MACP2-YFP* transgenic plants. Transcriptional level of *MACP2* in *MACP2-OE-1* and *MACP2-OE-2* upregulated 3–5 times as that in wild-type. The data represent means from three independent repeats. Statistical differences were identified using Student’s *t* test. ** *p* < 0.01. (**F**) Identification in protein level of *MACP2-YFP* transgenic plants. Anti-GFP was used to recognize the specific YFP tag. CBB represented Coomassie blue staining.

In *Arabidopsis* thaliana, besides the two reported proteins, NSL1 and CAD1, the function of the other two MACPF-containing proteins, MACP1 (encoded by At1g14780) and MACP2 (encoded by At4g24290), remain unknown. To explore the role of the MACP2 in *Arabidopsis thaliana*, we bought two T-DNA insertional mutants (Figure 1A, B) of this gene from TAIR and identified homozygotes. Amplification with primer pairs containing the T-DNA fragment LBa1 showed obvious bands but showed a blank with primer pairs for full-length MACP2 in mutants, which indicated that the mutants were exactly homozygous T-DNA insertion mutants. RT-PCR showed that no full-length transcripts were present in the corresponding *KO-1* and *KO-2* mutants (Figure 1C), indicating that these lines are knockout mutants. Subsequently, transgenic lines introducing the MACP2-YFP construct into the wild-type were generated at the same time. Clear bands of the vector contained fragment amplification results (Figure 1D) and higher relative expression levels of *MACP2* (Figure 1E); specific YFP-tagged MACP2 detection in *MACP2-YFP(OE)* plants via Western blot analysis (Figure 1F) showed the correct *MACP2-overexpression* transgenic plants we obtained.

### 2.2. Overexpression of MACP2-Accelerated Cell Death in Rosettes

**Figure 2:**
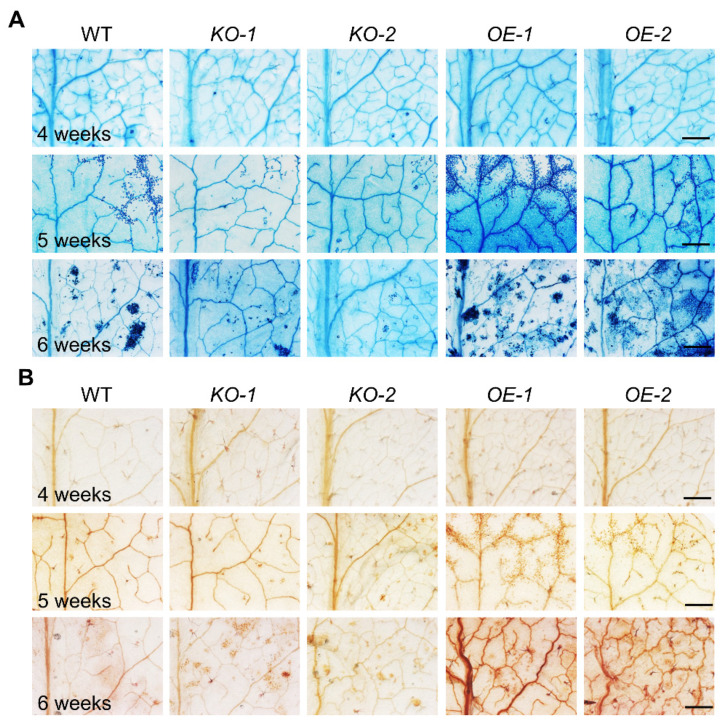
Overexpression of MACP2 showed accelerated cell death in rosettes. Trypan blue staining (**A**) and DAB staining (**B**) of wild-type, *MACP2-KO* mutants, and *MACP2-OE* rosettes after 4, 5, and 6-week development. *MACP2-OEs* obtained more cell death lesions and higher levels of H_2_O_2_, indicated by the brown color, than wild-type in the 5th and 6th weeks, while *MACP2-KOs* obtained less cell death and lower levels of H_2_O_2_ than wild-type. Bar = 1 mm.

To investigate whether disruption or overexpression of MACP2 shows linkage to SA, ROS accumulation, and constitutive cell death, we examined the rosettes of 4-, 5-, and 6-week-old wild-type, *KO* mutants, and *OE* transgenic plants using diaminobenzidine (DAB) and trypan blue staining. Interestingly, as shown in Figure 2, trypan blue-stained lesions displayed no significant differences at the fourth week and were apparently more severe in the leaves of *OE* transgenic plants than in the wild-type, whereas this was reversed in the *KO* mutants at the fifth and sixth weeks (Figure 2A). Meanwhile, *OE* leaves generated higher levels of H_2_O_2_ at the fifth and sixth weeks. This was indicated by the brown color upon DAB staining compared with the wild-type control rather than the lower level of H_2_O_2_ in the *KO* mutants than wild-type plants in the same period (Figure 2B). These results suggest that MACP2 promotes natural continuous cell death and ROS eruptions during leaf senescence.

### 2.3. MACP2-Strengthened Plant Resistance to Bacterial Pathogens Relying on the SA Pathway

**Figure 3:**
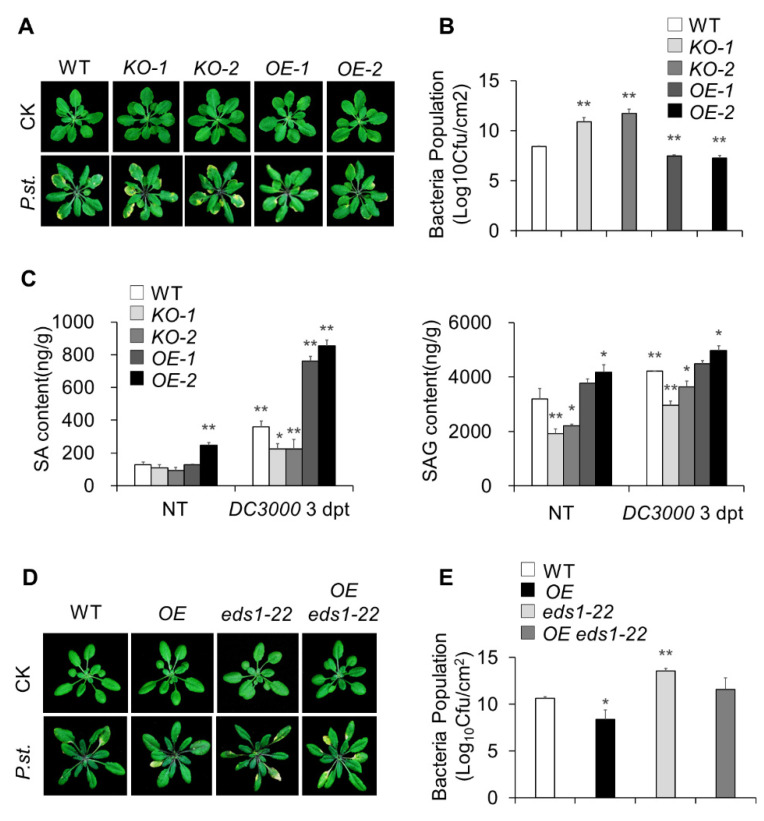
MACP2-strengthened plant resistance relying on SA pathway to the bacterial pathogen. (**A**) Phenotypes of wild-type, *MACP2-KOs*, and *MACP2-OEs* in response to *Pst* DC3000 infection. Four-week-old wild-type, *MACP2-KOs*, and *MACP2-OEs* were infected with *Pst* DC3000 on leaf surface and photographed 5 days after treatment. (**B**) Bacterial populations at 5 days postinoculation in wild-type, *MACP2-KOs*, and *MACP2-OEs* leaves. The data represent means from three independent repeats. Statistical differences were identified using Student’s *t* test. ** *p* < 0.01. (**C**) SA contents detection of wild-type, *MACP2-KO* mutants, and *MACP2-OEs* adult plants during *Pst* DC3000 infection. The contents of SA and SAG were measured by LC-MS. The “g” in “ng/g” represents the fresh weight. The experiments were biologically repeated three times with similar results. Error bars represent SD (*n* = 3 biological replicates). * *p* < 0.05, ** *p* < 0.01 by Student’s *t* test. (**D**) Phenotypes of leaves from 4-week-old wild-type, *MACP2-OE, eds1-22,* and *MACP2-OE eds1-22* leaves in response to *Pst* DC3000 infection. (**E**) Bacterial populations at 5 days postinoculation in wild-type, *MACP2-OE, eds1-22,* and *MACP2-OE eds1-22* leaves. The data represent means from three independent repeats. Statistically significant differences were identified using Student’s *t* test. * *p* < 0.05, ** *p* < 0.01.

To address whether the overexpression of MACP2 affects the plant defense response to bacterial pathogens, we conducted *Pst* DC3000 inoculation assays on four-week-old wild-type, *MACP2-KO* mutants, and *MACP2-OE* plants. According to the results, the *OE* plants showed a more tolerant phenotype than the wild-type when responding to *Pst* DC3000 infection (Figure 3A) and significantly repressed the bacterial population (Figure 3B), while the *KO* mutants showed a more sensitive phenotype than the wild-type (Figure 3A,B).

In addition, we measured the endogenous SA levels in wild-type, *KO* mutants, and *OE* transgenic lines during pathogen infection using liquid chromatography-mass spectrometry. As shown in Figure 3C, in the CK group, the contents of SA and SAG were higher in the *OE* plants but lower in the *KO* plants than in the wild-type (WT) plants. After *Pst* DC3000 treatment, although the SA content increased sharply in general, the SA and SAG contents in *OEs* were significantly higher than those in the wild-type. In contrast, a reverse trend of their content variation in response to *Pst* DC3000 treatment was found in the *KOs*.

On the basis of MACP2 promoting SA accumulation in response to bacterial pathogens, we selected enhanced disease susceptibility 1 (EDS1), forming heterodimers with phytoalexin deficient 4 (PAD4) to promote SA accumulation [31], and to generate *OE eds1-22* plants to further define the connection of the pathogen response and SA accumulation in *MACP2-OE* plants. Then, we conducted *Pst* DC3000 inoculation assays in four-week-old rosettes among different genotypes, including wild-type, *OE*, *eds1-22*, and *OE eds1-22*. Disrupting the SA signaling pathway EDS1 suppressed the resistance phenotype to *Pst* DC3000 in *OE* plants (Figure 3D,E). These results suggest that MACP2 strengthened plant resistance to bacterial pathogens depending on the SA pathway in Arabidopsis.

### 2.4. MACP2-Weakened Plant Resistance Depending on the SA Pathway to Necrotrophic Fungal Pathogens

**Figure 4:**
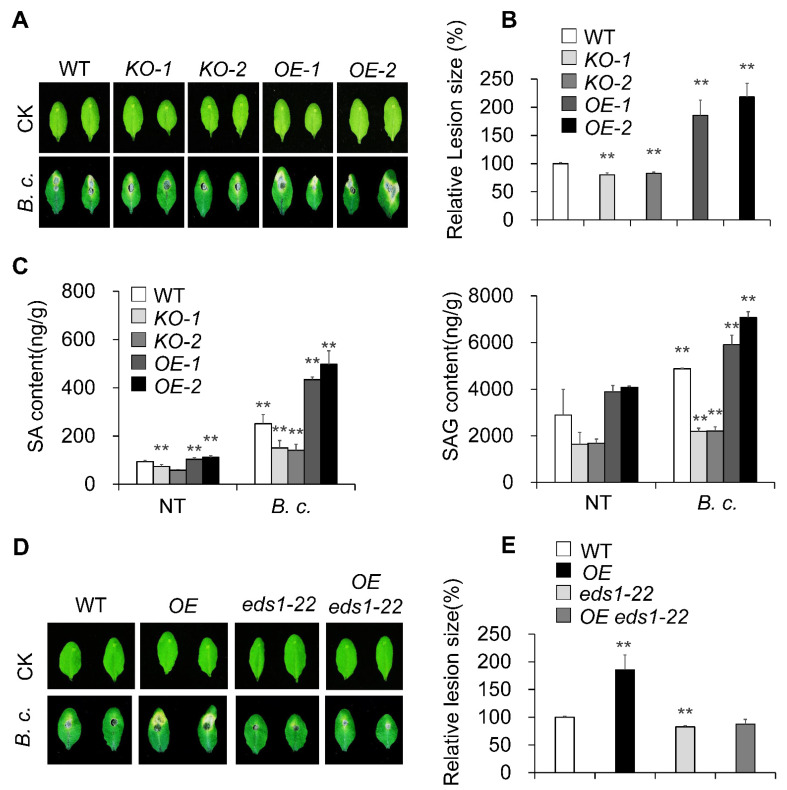
MACP2-weakened plant resistance depending on SA pathway to fungal pathogen. (**A**) Phenotypes of leaves from 4-week-old wild-type, *MACP2-KO* mutants, and *MACP2-OEs* plants in response to *B. cinerea* infection. Added *B.c.* on leaf surface and photographed 3 days after treatment. (**B**) Relative lesion size of wild-type, *MACP2-KOs*, and *MACP2-OEs* leaves after 3 days of *B. cinerea* infection. The lesion size was calculated by ImageJ and relative lesion size was calculated by comparing the values from treated leaves versus mock leaves. Asterisks indicate significant differences from the wild-type. ** *p* < 0.01 by Student’s *t* test. (**C**) SA contents detection of wild-type, *MACP2-KO* mutants, and *MACP2-OEs* adult plants during *B. cinerea* infection. The contents of SA and SAG were measured by LC-MS. The “g” in “ng/g” represented the fresh weight. The experiments were biologically repeated three times with similar results. Error bars represent SD. *n* = 3 biological replicates. ** *p* < 0.01 by Student’s *t* test. (**D**) Phenotypes of leaves from 4-week-old wild-type, *MACP2-OE, eds1-22,* and *MACP2-OE eds1-22* leaves in response to *B. cinerea* infection. (**E**) Relative lesion size of wild-type, *MACP2-OE, eds1-22,* and *MACP2-OE eds1-22* leaves after 3 days of *B. cinerea* infection. Asterisks indicate significant differences from the wild-type. ** *p* < 0.01 by Student’s *t* test.2.5. MACP2 Differentially Modulates Plant Sensitivities to Fungal and Bacterial Pathogens via the SA Signaling Pathway.

To explore whether the accumulation of SA in *OE* plants affected the response to necrotrophic fungal pathogens, we conducted *B. cinerea* inoculation assays on four-week-old wild-type, *KO* mutant, and *OE* plants. *OE* plants were hypersensitive to *B. cinerea* infection compared to wild-type plants (Figure 4A,B), as confirmed by the larger lesion size in *OE* plants instead of the resistant phenotype, and the smaller lesion size in *KO* plants (Figure 4A, B). Naturally, we also tested the contents of SA and SAG that were also induced during fungal infection and accumulated in *OEs* (Figure 4C). *B. cinerea* infection experiments were also carried out on *OE eds1-22* plants and revealed that the absence of the SA signal pathway EDS1 crippled the sensitive phenotype to *B. cinerea* in *OE* plants (Figure 4D,E). These results suggest that MACP2 operates differentially in response to bacterial and necrotrophic fungal pathogens.

**Figure 5:**
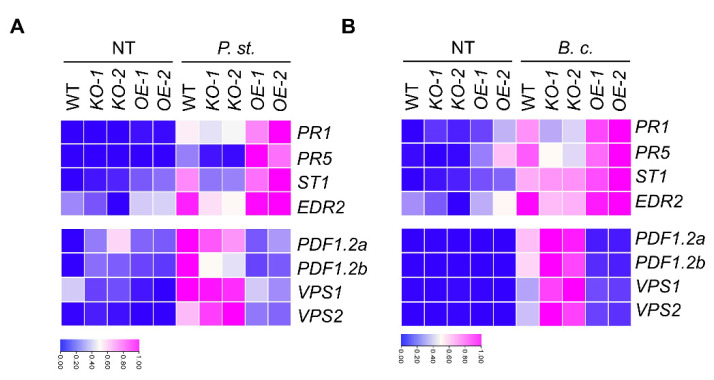
MACP2 differentially modulate plant sensitivities to fungal and bacterial pathogens via SA signaling pathway. Heatmaps show the fold change of key regulators in SA and JA signaling pathways in wild-type, *MACP2-KO* mutants, and *MACP2-OEs* plants after infection with *Pst* DC3000 (**A**) and *B. cinerea* (**B**). The transcriptional profiles of relative gene expression values were analyzed using the TB tools.

To further investigate the difference between bacterial and fungal immunity caused by *MACP2* overexpression, we detected the expression of genes related to SA-associated defense responses, including *PR1*, *PR5*, *ST1*, and *EDR2* [32,33,34], and JA-associated defense responses, including *PDF1.2a*, *PDF1.2b*, *VPS1*, and *VPS2* [35] in rosettes. Consistent with the phenotype resistant to bacterial pathogen in *OE* plants, the transcript levels of SA-associated responsive genes in *OE* plants (with the exception of *EDR2*) showed a sharply upregulated trend compared with the wild-type but displayed a significantly downregulated trend in *KO* mutants. On the contrary, an inverse trend was observed for JA-related responsive genes (Figure 5A), manifesting in significant upregulation in *KO* mutants but downregulation in *OEs.* With the necrotrophic fungal pathogen *(B. cinerea)*, the *OEs* displayed downregulation of JA-responsive genes, and *KOs* showed fiercely higher expression than the wild-type. In addition, SA-responsive genes were also induced slightly by *B. cinerea* and maintained a higher expression level in *OEs,* resulting in a fungus-sensitive phenotype based on the antagonizing role of SA and JA. Our results suggest that overexpression of MACP2 may contribute to accelerated SA accumulation, thus activating the SA signaling pathway in response to pathogen invasion.

### 2.5. Alternatively Spliced Isoforms of MACP2 Are Differentially Expressed under Pathogen Treatment

**Figure 6:**
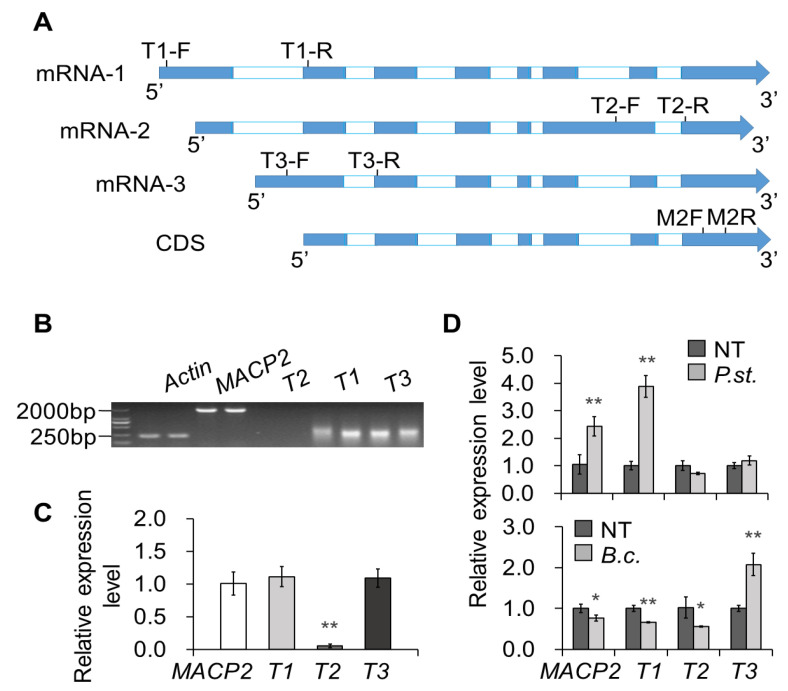
AS of MACP2 responded to fungal and bacterial pathogens. (**A**) Classification of three AS of MACP2 gene. The blue represents exons and white represents introns. (**B**) Semiquantitative PCR detection of full-length MACP2 and specific sequence of each AS (T1, T2, T3) in 4-week-old rosettes of wild-type. The full-length of MACP2 was amplified with primer pair XS2591 and XS2592. The specific sequences of AS were amplified with primer pairs MACP2-1 F/MACP2-1 R, MACP2-2 F/MACP2-2 R, and MACP2-3 F/MACP2-3 R, respectively. (**C**) qRT-PCR detection of common sequence (MACP2) and specific sequence of each AS (T1, T2, T3) in 4-week-old rosettes of wild-type. The common sequence of three AS was amplified with primer pair MACP2-F and MACP2-R. The specific sequences of AS were amplified with primer pair mentioned in (**B**). The *ACTIN2* was amplified with primer pair ACTIN2-F and ACTIN2-R. Asterisks indicate significant differences from the wild-type. ** *p* < 0.01 by Student’s *t* test. (**D**) qRT-PCR detection of AS responding to *Pst* DC3000 and *B. cinerea* after 3 days of infection on 4-week-old rosettes of wild-type. Asterisks indicate significant differences from the wild-type. * *p* < 0.05, ** *p* < 0.01 by Student’s *t* test.

To investigate the relationship between AS regulation of MACP2 and pathogen resistance, we designed isoform-specific primers to explore the expression levels of these isoforms either under normal conditions or treatment with *Pst* DC3000 or *B. cinerea* (Figure 6A). A total of three mRNAs can be detected for MACP2, named *MACP2-1*, *MACP2-2*, and *MACP2-3*. Expression analysis using semiquantitative and real-time quantitative PCR indicated that *MACP2-1* and *MACP2-3* were highly expressed in four-week-old rosettes of the wild-type before any treatments (Figure 6B, C). Interestingly, *Pst* DC3000 induced the expression of the *MACP2* locus, particularly through the transcription of *MACP2-1*, but not the other two mRNA isoforms. In contrast, the transcript abundance of *MACP2* was reduced in response to *B. cinerea* inoculation. Isoform-specific expression analysis suggested that *MACP2-1* and *MACP2-2* contribute to this reduction, whereas *MACP2-3* was elevated in comparison to untreated controls. Thus, the underlying mechanism of this differential expression of MACP2 isoforms in response to different pathogens remains to be further investigated.

### 2.6. Indolic GS Contributed to Bacterial Resistance to MACP2-OE

**Figure 7:**
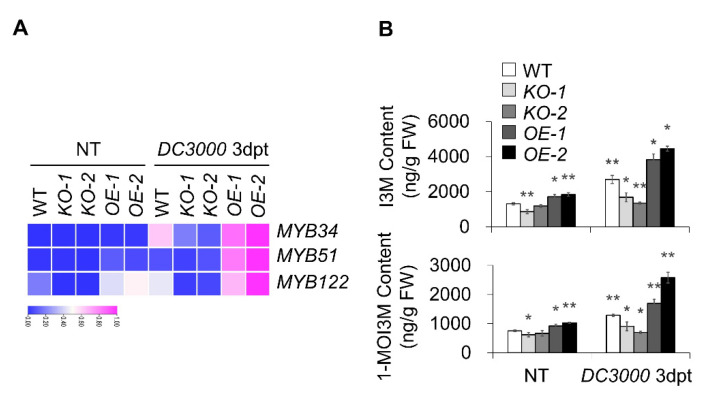
Indolic GS contributed to bacteria resistance of *MACP2-OE*. (**A**) Heatmaps show the fold change of key regulators in GS biosynthesis, containing *MYB34*, *MYB51*, and *MYB122* in wild-type, *MACP2-KO* mutants, and *MACP2-OEs* plants after *Pst* DC3000 infection. The transcriptional profiles of relative gene expression values were analyzed using the TB tools. (**B**) Indolic GS contents of wild-type, *MACP2-KO* mutants, and *MACP2-OEs* plants after *Pst* DC3000 infection. The contents of indolic GS were measured by LC-MS. The experiments were biologically repeated three times with similar results. Error bars represent SD. *n* = 3 biological replicates. Asterisks indicate significant differences from the wild-type. * *p* < 0.05, ** *p* < 0.01 by Student’s *t* test.

To explore whether the increased SA contents in *MACP2-OEs* are related to tryptophan (Trp)-derived glucosinolates, we examined the transcription level of six vital regulators in GS biosynthesis in *Arabidopsis* plants, including MYB34, MYB51, MYB122 (involved in the synthesis of indolic GSs), MYB28, MYB29, and MYB76 (related to the synthesis of aliphatic GSs), and the level of GS contents, including the indolic GSs indol-3-ylmethyl-GS (I3M) and 1-methoxyindol-3-ylmethyl-GS (1-MOI3M), and the aliphatic GSs 4-methylsulfinylbutyl-GS (4-MOSB), 5-methylsulfinylpentyl-GS (5-MSOP), and 8-methylsulfinyloctyl-GS (8-MSOO) in wild-type, *MACP2-KO* mutants, and *MACP2-OE* plants rosettes after 3 days of *Pst* DC3000 infection. The levels of indolic GS synthesis regulators (MYB34, MYB51, and MYB122) and indolic GS species (I3M and 1-MOI3M) in response to *Pst* DC3000 infection were significantly elevated in *OE* plants but significantly reduced in *KO* mutants (with the exception of *MYB51*) compared to the wild-type (Figure 7A, B). In contrast, the transcriptional level of aliphatic GS synthesis regulators (MYB28, MYB29, and MYB76) and the levels of aliphatic GSs, including 4-MOSB, 5-MSOP, and 8-MSOO, were not significantly altered in *OEs*, while those related to aliphatic GSs and the levels of aliphatic GSs increased in response to *Pst* DC3000 infection in general (Supplementary Figure S1). These findings indicate that MACP2 responds to pathogen infection in a tryptophan (Trp)-derived indole glucosinolate-activated SA-dependent manner.

## 3. Discussion

### 3.1. Pleiotropic Function of MACPF Proteins in Plant Immunity and Programmed Cell Death

An orthologue search indicated that there are four MACPF proteins in Arabidopsis, and two of them have been extensively studied in the past twenty years [19]. In this study, we demonstrated that *macp2* knockout mutants and *MACP2-OE*s display altered sensitivity to bacterial and fungal pathogens (Figure 3 and Figure 4), suggesting that MACP2 participates in plant immunity responses to external pathogens. Interestingly, the knockout mutant of *CAD1* showed similar lesion mimic phenotypes [10,15,36], suggesting the activation of immune responses in this knockout mutant. Previous genetic and physiological studies have demonstrated that the *cad1-1* mutant is resistant to the virulent bacterial pathogen *Pst DC3000* [15]. Similarly, the *MACP2-OEs* had a similar phenotype (Figure 3), showing fewer lesions in comparison to the wild-type. The MACP2 shared 52.1% and 43.9% identity with NSL1 and CAD1, respectively [15]. The underlying mechanism of phenotypic variation among these three MACPFs remains to be further investigated. One hypothesis that has been proposed previously is that nonoverlapping functions of NSL1 and CAD1 may be related to downstream defense-related R proteins [30]. At the molecular level, the *cad1-5* mutant has been found to elevate *PR1* gene expression, the marker of plant immunity [15]. However, this hypothesis needs direct experimental evidence for further investigation.

Furthermore, the expression of NSL1 and CAD1 is not induced by biotic stress treatments but is altered under abiotic stress conditions, suggesting that constitutive defense responses of these Arabidopsis mutant lines may not be the primary function of these MACPF proteins [30]. Indeed, the massive production of reactive oxygen species (ROS) through oxidative bursts during plant-pathogen interactions will trigger PCD in plants [37]. NSL1 has been proposed to disturb ROS production, thus impairing PCD during plant–disease responses [30], whereas overexpression of MACP2 caused higher levels of H_2_O_2_ and profound cell death in rosette leaves (Figure 2).

In mammals, to form a transmembrane pore structure, MACPF domain-containing proteins require the assisted assembly of other complement proteins [38,39]. However, no secretory peptide signal could be detected in the protein sequence of *Arabidopsis* MACPFs [15]. A previous study of NSL1 suggested that the metabolic imbalance detected in the *nsl1* mutant may be the result of improper assembly of these pore structures [30]. By using 35S- and native promoter-driven constructs, NSL1 was found to localize at the plasma membrane in Arabidopsis. Similarly, the subcellular localization of CAD1 has been confirmed by fractionation and confocal microscopy approaches [15]. In this study, MACP2 is a membrane-localized protein (Supplemental Figure S2), indicating that all three MACPFs can be deployed by the plant immune system to the entry site as a defense mechanism during host–microbe interactions. Unfortunately, there is little hard experimental evidence to prove the formation of protein complexes by these MACPFs in plants. Our previous study characterized *MACPF* genes in plants and revealed that several of those in Poaceae participated in plant vegetative growth and environmental stress adaptation [18]. In addition, nonredundant phenotypes of CAD1 and NSL1 suggested that plant MACPFs may function differently from their animal counterparts by assembling heteromeric complexes themselves to create pore structures on cell membranes [15]. Furthermore, although NSL1 is localized at the cell membrane, it did not kill pathogens at the entry site, suggesting that NSL1 has a differential mechanism in comparison to their animal orthologues. However, further molecular and biochemical experiments are required to unravel the underlying mechanism of plant MACPFs.

### 3.2. Plant Hormonal Signaling Is Critical to Influence MACP2-Mediated Disease Resistance

Plant hormones are important for all aspects of plant growth and physiology [40,41,42,43]. To unravel the molecular mechanism of MACP2-mediated PCD in plant immune responses, the relationship between plant defense hormones and MACP2 were evaluated. Genetic analysis of transgenic plants suggested that MACP2-mediated PCD is dependent on the plant hormone SA (Figure 3), which is similar to the molecular mechanism of CAD1 [15]. It has long been reported that the elimination of SA content could inhibit the expression of *PR* genes and thus lower resistance to pathogen infection [44]. The *cad1-1* mutant has a higher level of SA content than the wild-types, and the introduction of the bacterial enzyme NahG for SA degradation could rescue the PCD phenotype of *cad1-1* [15]. Meanwhile, the SA content increased significantly after treatment with both bacterial (*Pst DC3000*) and fungal pathogens (*B. cinerea*).

Furthermore, NSL1-mediated PCD triggered by flg22 has been considered a potential PAMP response and is characterized by the accumulation of SA and ROS, which are typical MTI outputs in response to pathogen attacks [4,45]. In contrast, CAD1 has been proposed to induce HR-related cell death by activating NLR signaling [36,46]. In this article, subsequent analysis indicated that EDS1 is downstream of MACP2 to confer plant immune responses (Figure 3 and Figure 4). Similarly, most of the phenotypic and biochemical changes among CAD1 transgenic lines are proposed to be dependent on EDS1-mediated signaling [15], and approximately 90% of SA biosynthesis in plants is affected by EDS1-PAD4 signaling in the cytosol and nucleus [47,48]. EDS1 is a nucleocytoplasmic lipase-like protein that is classified as a member of the NLR-TNL signaling pathway by forming heterodimers with either phytoalexin deficient 4 (PAD4) or SAG101 [12,13]. Furthermore, the *nsl1-3 pad4* double mutant did not show a hyperactive immunity phenotype, indicating that NSL1 is guarded by NLR-TNL signaling [4]. In addition to SA biosynthesis activation, the EDS1-PAD4 complex is able to induce the expression of genes involved in the cell death response, such as *PR1*. In this study, the expression of *PR1* and *PR5* among transgenic Arabidopsis *MACP2-OEs* correlated with SA levels, further validating that EDS1 is responsible for MACP2-mediated PCD. Nevertheless, except for EDS1, downstream signaling of MACPFs in response to plant pathogens remains to be further investigated.

Intriguingly, a recent report demonstrated that the EDS1-PAD4 pair participates in sphingolipid metabolism to trigger cell death in response to the fungal pathogen *B. cinerea* [13]. The involvement of sphingolipids, especially long-chain ceramides, in the MACP2-mediated PCD response is valuable for study. Furthermore, SA resists the biotrophic pathogens living and reproducing on live host cells, whereas jasmonic acid (JA) acts on necrotrophic pathogens that kill host cells for nutrition and reproduction. Both of them play important but antagonistic signaling roles in pathogen responses [49]. EDS1-PAD4 signaling has been reported to play a negative role in response to *B. cinerea*, a necrotrophic fungal pathogen that can activate the JA pathway in plants [50]. Thus, the phenotypes of MACP2 in response to *B. cinerea* could be explained (Figure 4), suggesting that the repression of cell death in Arabidopsis effectively confers plant resistance to *B. cinerea*. Similarly, JA accumulated in the *cad1* mutant, and the JA/ethylene-induced gene *PDF1.2* was altered compared to wild-types [10]. Specifically, *PDF1.2a* and *PDF1.2b* were differentially expressed in MACP2 transgenic lines in response to bacterial and fungal pathogens (Figure 5), suggesting crosstalk between multiple plant hormonal signaling pathways downstream of MACP2. Finally, different splice isoforms responded to bacterial or fungal inoculation (Figure 6), indicating that the distinct response mechanism of MACP2 to bacterial and fungal pathogens can be controlled by posttranscriptional regulation, i.e., alternative splicing [51,52,53,54,55].

### 3.3. Glucosinolates Are Crucial Signal Messengers That Transduce Immunity-Triggered PCD Downstream of MACPF Proteins

Previously identified EDS1-PAD4 signaling has been documented as a universal regulator of plant immunity, which also regulates multiple metabolic pathways of plant hormones, phytoalexins (camalexin), and other secondary metabolisms (tocopherols and N-hydroxypipecolic acid) [56]. In the study of NSL1, glucosinolates (GSs), an unsuspected role of tryptophan-derived secondary metabolites, are pivotal messengers to initiate PCD by activating SA biosynthesis in Arabidopsis [4]. Glucosinolates (sulfur- and nitrogen-containing thioglucosides) show broad activity against insect herbivores and plant pathogens [57] and are classified into three subcategories: aromatic GSs, methionine-derived aliphatic GSs (AGSs), and tryptophan-derived indole GSs (IGSs) [58].

A pathogen-inducible myrosinase, penetration 2 (PEN2) involved in the bioconversion of indole glucosinolates (IGSs) [59], plays an important role in PAMP-triggered PCD in the absence of NSL1 [4]. PEN2 is responsible for releasing bioactive molecules (e.g., isothiocyanates) with a wide range of toxicity to insects and plant pathogens [60]. In particular, 4-methoxyindol-3-ylmethylglucosinolate (4MI3G, IGS against a broad spectrum of fungal pathogens) is accumulated [61] under pathogen infection via PEN2 activity. In our study, indolic GS species (I3M and 1-MOI3M) were highly accumulated in *MACP2-OEs* and were less accumulated in *MACP2-KOs* in comparison to the levels of these compounds in the wild-type plants (Figure 7), implying that IGs may function similarly as signal molecules to connect MACP2 and downstream PCD responses. Furthermore, the conversion of I3G to 4MI3G has been proposed to be tightly regulated by the mitogen-activated protein kinase (MAPK)-transcription factor (TF) cascade. The MPK3/MPK6-MYB34/51/122 cascade has been suggested to participate in this regulation [62]. Here, the transcript abundance of three R2R3-MYB TFs, *MYB34*, *MYB51*, and *MYB122,* was tested, showing a high correlation with the content of I3M and 1-MOI3M in *MACP2-KOs* and *MACP2-OEs* (Figure 7). However, the mechanism by which the EDS1-PAD4 pair triggers IGS biosynthesis remains elusive. Further study of signal transduction downstream and the assembly mechanism of MACP2 will be informative because this is the general defense mechanism that plants possess to restrict pathogen infection.

## 4. Materials and Methods

### 4.1. Plant Material, Growth Condition, and Treatment

*Arabidopsis thaliana* accession Columbia-0 (Col-0) was used as the wild-type line in this study. *MACP2-KO-1* (SALK_040186) and *MACP2-KO-2* (SALK_052845C) were obtained from The Arabidopsis Biological Resource Center (ABRC, USA, http://www.arabidopsis.org, accessed on 24 April 2015). The *eds1-*22 mutant used in this study has been described previously [63]. For genetic analysis, the *eds1-22* mutant was crossed with *MACP2-OE* to generate *OE eds1-22*. For the seed germination assay, the Arabidopsis seeds were surface sterilized with 20% bleach containing 0.1% Tween 20 (Sigma, P2287, St. Louis, MI, USA) for 15 min, washed with distilled water 6 times, and then plated on 1/2 MS (Sigma, M5519, USA) agar with 1% sucrose. The plates were incubated at 4 °C for 2 days and then transferred to a greenhouse under a 16 h light/8 h dark photoperiod at 20 °C for 7 days according to a previous study [64].

### 4.2. Plasmid Construction and Transgenic Plant Generation

All constructs were generated using the ClonExpress II One Step Cloning Kit (Vazyme, C112, China). The gene-specific primers with 15 bp extensions homologous to the corresponding vectors are listed in Supplemental Table S1.

To generate stable transgenic plants, MACP2 CDS was cloned into pUC119-YFP to construct the expression cassette MACP2-YFP, which was cloned into the binary vector pFGC-RCS via the same *Asc*I digestion site between the two vectors [65,66]. The expression cassettes were subsequently introduced into wild-type Arabidopsis (Col-0) by Agrobacterium tumefaciens-medium transformation via the floral dip method [67] to generate *MACP2-YFP* transgenic plants.

### 4.3. DAB and Trypan Blue Staining

Trypan blue staining and DAB staining were performed according to Xiao and Chye [68]. For trypan blue staining, rosettes of 4-, 5-, and 6-week-old wild-type, *MACP2-Kos*, and *MACP2-OE* plants were collected in 10 mL tubes and boiled for 5 min in trypan blue staining buffer composed of 12.5% phenol (Thermo, K2599312, Waltham, MA, USA), 12.5% glycerol (Guangzhou Chemical Reagent Factory, Guangzhou, China), 12.5% lactic acid, 48% ethanol (Guangzhou Chemical Reagent Factory, China) and 0.025% trypan blue. The rosettes were incubated for 10 min at room temperature and then decolorized five times in 70% chloral hydrate. For DAB staining, rosettes of 4-, 5-, and 6-week-old wild-type, *MACP2-Kos*, and *MACP2-OE* plants were collected and incubated in 1 mg/mL DAB (Sigma, D8001, USA) solution of 10 mM PBS (Vazyme, G101, pH 7.0, Nanjing, China) and 0.05% Tween 20 at 37 °C in darkness overnight and subsequently decolorized in 95% ethanol at 65 °C 3 times every 2 h.

### 4.4. Pathogen Infection

Pathogen inoculation was carried out as previously described [69,70,71] with minor modifications. The fungal pathogen *Botrytis cinerea* was maintained on V8 juice agar medium at 25 °C in the dark for 10 days. Spore masses were collected and suspended in Vogel buffer composed of 50 mM sucrose (Guangzhou Chemical Reagent Factory, China), 20 mM K_2_HPO_4_ (Damao Chemical Reagent Factory, China), 10 mM sodium citrate, 20 mM (NH_4_)_2_SO_4_, 1 mM MgSO_4_ and 10 mM CaCl_2_ (pH 5.0). More than 9 mature rosettes per genotype from different 4-week-old plants were placed in petri dishes containing 0.6% (*w*/*v*) agar. Each leaf was inoculated with 5 μL droplets containing 1.6 × 10^6^ spores/mL of *B. cinerea* suspension, incubated in the dark for 36 h, and then cultivated in a greenhouse with a 16 h light/8 h dark photoperiod at 20 °C. The lesion diameter (mm) was calculated using ImageJ software.

The bacterial pathogen *P. syringae* pv. *tomato* (*Pst* DC3000) was cultivated at 28 °C and 200 rpm in King’s medium B [70] containing rifampicin (New Probe, 50 mg/L, China). Then, *Pst* DC3000 was collected by centrifugation and resuspended in 10 mM MgCl_2_ at A_600_ = 0.2. Bacteria were then diluted 10 times to approximately 107 colony-forming units/mL in 10 mM MgCl_2_ and 0.02% Silwet L-77 (New Probe, P001374, China) for inoculation. After inoculation, the plants were kept in high humidity. To calculate the bacterial populations, leaf discs (0.6 cm diameter) were collected from infected leaves, washed three times with sterile water, and homogenized in 10 mM MgCl_2_, followed by applying appropriate dilutions on solid King’s B medium with rifampicin. All experiments were repeated three times with similar results.

### 4.5. SA Measurements

SA was extracted and measured as described previously [72,73]. Approximately 150 mg powdered tissue was weighed in a 2 mL centrifuge tube and extracted with 800 μL of extraction buffer of 2-propanol/water/concentrated HCl (2:1:0.005, *v*/*v*/*v*) with internal standards of 10 ng d4-SA (Sigma-Aldrich, USA). The mixtures were shaken mildly for 30 min at 4 °C, followed by adding 1 mL dichloromethane and shaking for an additional 30 min at 4 °C. The samples were then centrifuged at 13,000× *g* and 4 °C for 10 min. Solvent (1 mL) from the lower phase was collected and dried using a nitrogen evaporator with nitrogen flow. The samples were dissolved in a 200 μL mixture of 60% methanol (Mreda, M042749, China) and 40% sterile ultrapure water. Quantitative analysis of SA was performed via a chromatography (Shimadzu, Japan)–mass spectrometry (Triple TOF 5600, AB SCIEX, USA) system according to Chen et al. [72].

### 4.6. RNA Extraction and RT-qPCR

Total RNA was extracted from 5- and 6-week-old *Arabidopsis* leaves referring to a previous study [35]. Two milligrams of total RNA were extracted by HiPure Plant RNA Mini kit (Magen, China) and converted into cDNA with the HiScript II QRT Super Mix kit with gDNA Wiper (Vazyme). RT-qPCR assays (10 μL reaction volumes with gene-specific primers, Supplemental Materials Table S1) were performed on a StepOne Plus Real-time PCR System (Applied Biosystems) using ChamQ SYBR Color qPCR Master Mix (Vazyme, China) and the following protocol: 95 °C for 5 min followed by 40 cycles of 95 °C for 15 s, 55 °C for 15 s, and 72 °C for 30 s.

Primers for RT-PCR were described in both a previous publication and qPrimer DB (https://biodb.swu.edu.cn/qprimerdb/, accessed on 24 April 2015, [74]). Primers for specific AS were designed in exon–exon junction, for which specificity was verified via Primer-Blast software and amplified in restricted extension time to tule out genomic DNA contribution. The efficiency of each primer pair was not evaluated and only comparisons for each particular mRNA isoform under normal conditions or pathogen treatment were compared to draw further conclusions. For calculation of relative transcription levels, the delta of threshold cycle (∆Ct) values was calculated by subtracting the arithmetic mean Ct values of the target genes from the normalizing *ACTIN2*. The relative transcription level (2^∆∆Ct) was calculated from three independent experiments. The fold change values were visualized, illustrated, and standardized in a heatmap generated by the TBtools package [75]. In the heatmap, the color represents the fold change value. The closer it is to pink, the greater the fold change value.

### 4.7. GS Measurements

GSs were extracted and detected as described previously [35,76] with minor modifications. Frozen leaf samples (120 mg) were ground with a glass rod in 1.2 mL ice-cold MeOH/H_2_O (70:30, *v*/*v*) and incubated at 80 °C for 15 min. The homogenate was centrifuged at 3500× *g* and 4 °C for 10 min, and the supernatant was filtered through a 0.22 μm filter for analysis. Chromatography (Shimadzu, Japan)–mass spectrometry (Triple TOF 5600, AB SCIEX, USA) was used to detect and analyze the GS contents according to Liao et al. [35]. Quantification was performed using three technical replicates. Experiments were repeated three times with similar results, and five plants of each genotype were collected for one technical replicate.

### 4.8. Protein Isolation and Immunoblot Analysis

For total protein extraction, 4-week-old *Arabidopsis* seedlings grown in soil were ground in liquid nitrogen and homogenized in ice-cold protein extraction buffer of 50 mM sodium phosphate (pH 7.0), 200 mM NaCl, 10 mM MgCl_2_, 0.2% β-mercaptoethanol (Westgene, WG0482, China), and 10% glycerol, and supplemented with protease inhibitor cocktail (Roche, 04693132001) according to Xia et al. [77]. The samples were placed on ice for 30 min and centrifuged at 4 °C at 12,000× *g* for 10 min. The supernatant was transferred to a new microfuge tube before electrophoresis.

For immunoblot analysis, clarified extracts were subjected to SDS-PAGE and transferred to a Hybond-C membrane (Cytiva, 10600002, USA). Specific anti-GFP (Abmart, M20004S, 1:5000, China) antibody was used in the protein blotting analysis.

### 4.9. Statistical Analysis

The significance of the difference between 2 groups was determined using Student’s *t* test. The level of statistical significance is indicated by asterisks (* *p* < 0.05 and ** *p* < 0.01). The numbers of samples are indicated in the figure legends.

## 5. Conclusions

Collectively, this study reveals the molecular mechanism of the Arabidopsis MACPF domain-containing protein MACP2 in the plant immune response. The natural PCD, bacterial pathogen resistance and necrotrophic fungal pathogen sensitivity observed in *MACP2-OEs* is possibly mediated by the activation of IGSs and endogenous SA biosynthesis through the EDS1 signaling pathway. These findings provide a genetic framework and knowledge base to study the biochemical function of plant MACPF proteins in future works.

## Data Availability

Not applicable.

## Supplementary Materials

The following supporting information can be downloaded at: https://www.mdpi.com/article/10.3390/ijms23158784/s1.
