# Supplementary material for: Overexpression of the Arabidopsis MACPF Protein AtMACP2 Promotes Pathogen Resistance by Activating SA Signaling
Source: Int J Mol Sci. 2022 Aug 7;23(15):8784. doi: 10.3390/ijms23158784 (PMC9369274; doi:10.3390/ijms23158784)
Supplement: Supplementary file 1 [file ijms-23-08784-s001.zip › ijms-1830202-supplementary.pdf]

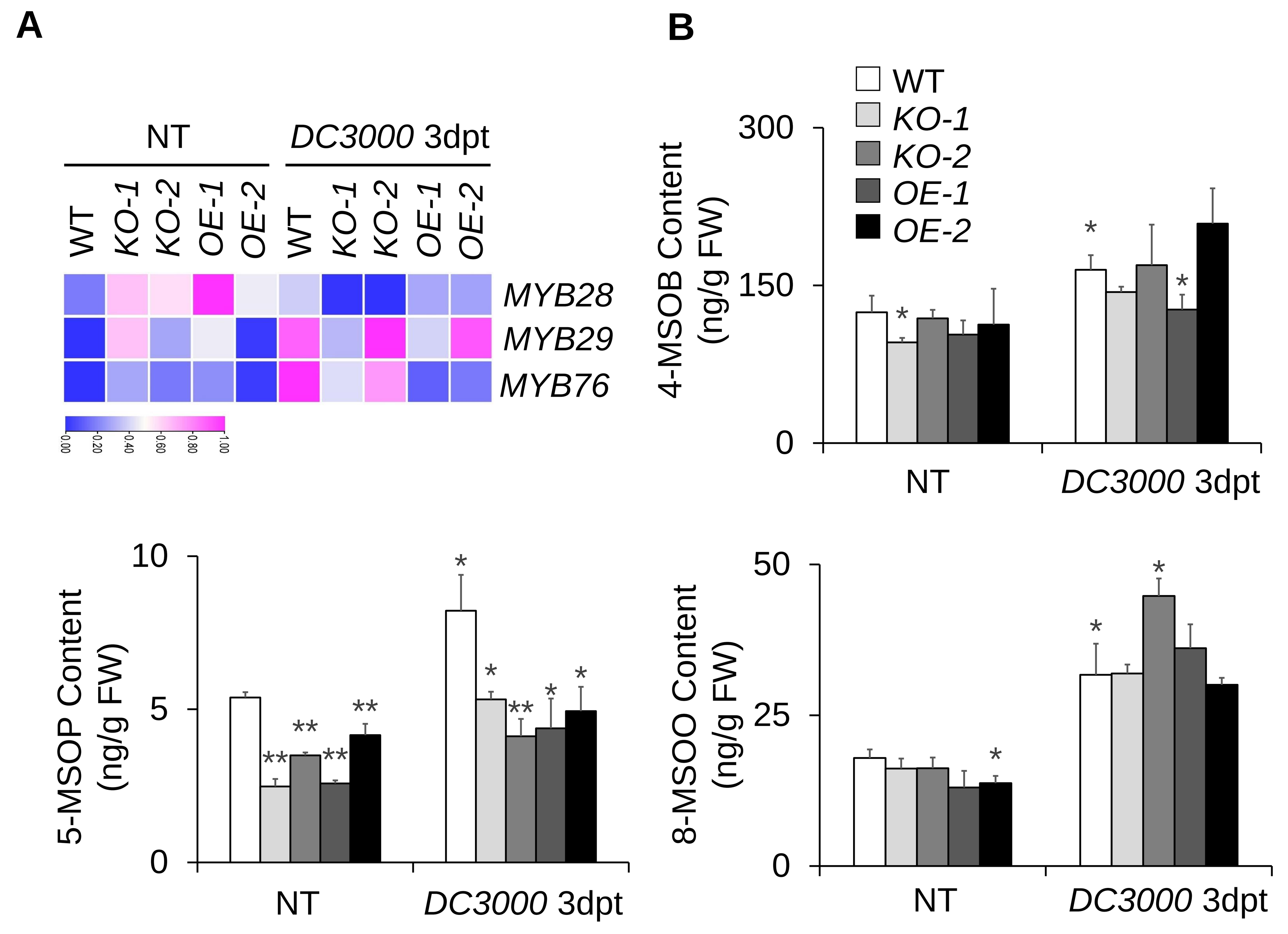

Supplemental Figure S1. Contents of aliphatic-GS in wild type, MACP2-KOs, and MACP2-OEs.

(A) Heatmaps show the fold change of key regulators in GS biosynthesis, containing MYB28, MYB29, and MYB76 (involved in the synthesis of aliphatic GSs) (|log2 fold change| > 1) in wild-type, MACP2-KO mutants and MACP2-OEs plants after with P.st. DC3000 infection. Red and blue represent up-regulated and down-regulated genes, respectively. The transcriptional profiles of relative gene expression values were analyzed using the TB tools.

(B) Aliphatic GS contents detection of wild-type, MACP2-KO mutants and MACP2-OEs plants after P.st. DC3000 infection. LC-MS measurements showed that contents of aliphatic GSs including 4-MOSB, 5- MSOP, and 8-MSOO were not significantly altered in OEs after 3-d-infection of P.st. DC3000. The experiments were biologically repeated three times with similar results. Error bars represent SD (n = 3 technical replicates). (**P < 0.01 by Student’s t test).

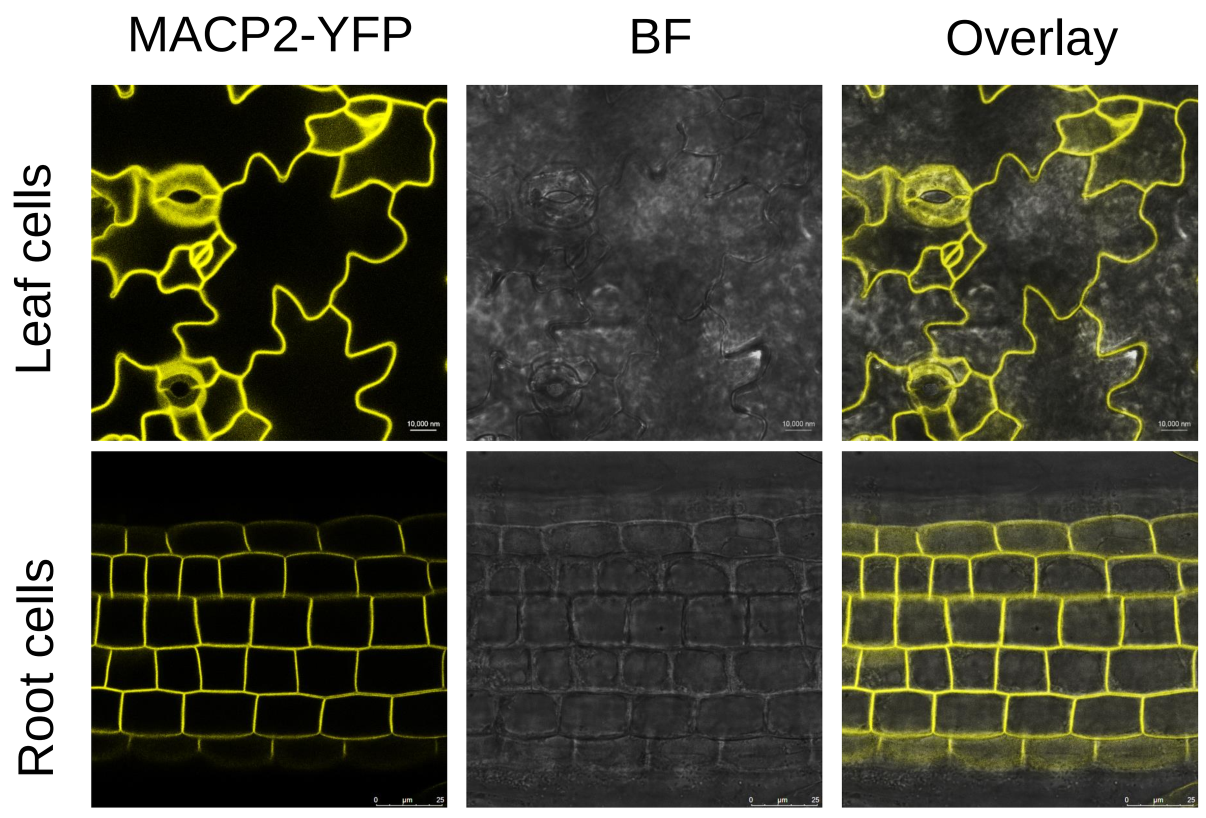

Supplemental Figure S2. MACP2 protein is subcellular localized at the plasma membrane.

Localization of MACP2-YFP in the leaf and root of transgenic plants. YFP was excited at 514 nm and the emissions were collected using a 554/30 band-pass emission filter on Leica SP8X. The scale on the images of leaf and root are 10 µm and 25 µm respectively.

Table 1. Primers Used for Identification, Vector Construction and Quantitative Real-time qPCR Analysis

Name	Description	Sequence(5'-3')
XS2591<br>Mutant, *OE* Characterization	Forward	GCTCTTCGGCTACCAGCTTCTAAAG
XS2592<br>Mutant Characterization	Reverse	GAGATTTTGCCTTTCTCCAC
XS2489<br>*OE* Characterization	Reverse	CCATCTGAACTGTTTGCAGC
pFGC-MACP2-YFP<br>vector construction for *OE*	Forward	TGATTAACAGGGATCCATGGCTCTTCGGCTACC
pFGC-MACP2-YFP<br>vector construction for *OE*	Reverse	AATGTTTGAACGATCTGCAGAGATCTGTACAGCTCCTCC
MACP2-Realtime qPCR	Forward	GCCTGTCGAATCAGATGATG
MACP2-Realtime qPCR	Reverse	CTTTACCAGTGTTGCTCCCAC
Actin2-Realtime qPCR	Forward	CTTGCACCAAGCAGCATGAA
Actin2-Realtime qPCR	Reverse	CCGATCCAGACACTGTACTTCCTT
PDF1.2a-Realtime qPCR	Forward	TTTGCTGCTTTCGACGCAC
PDF1.2a-Realtime qPCR	Reverse	TAACATGGGACGTAACAGATA
PDF1.2b-Realtime qPCR	Forward	CTTTGCAGCTTTTGAAGTACC
PDF1.2b-Realtime qPCR	Reverse	GCTCCTTCAAGGTTAATGCACT
VPS1-Realtime qPCR	Forward	GGCGTACTGGTCGTGGTTAGAGT
VPS1-Realtime qPCR	Reverse	AGCCTTGAGATTCTCGACAGTGACT
VPS2-Realtime qPCR	Forward	TCAGTGACCGTTGGAAGTTGTG
VPS2-Realtime qPCR	Reverse	GTTCGAACCATTAGGCTTCAATATG
PR1-Realtime qPCR	Forward	TGGTCACTACACTCAAGTTGTT
PR1-Realtime qPCR	Reverse	GCTTCTCGTTCACATAATTCCC
PR5-Realtime qPCR	Forward	AGGATTTGAATTGACTCCAGGT
PR5-Realtime qPCR	Reverse	CCATCGCCTACTAGAGTGAATT
ST1-Realtime qPCR	Forward	GGCACACACAAGCTATTTTACA
ST1-Realtime qPCR	Reverse	TTAGTGACGAGGATAATGTCGG
EDR2-Realtime qPCR	Forward	GAAAGAACTAGTACCTGGGTCC
EDR2-Realtime qPCR	Reverse	AATTTCTAAGTATGTCGGGCCA
MACP2-1 Realtime qPCR	Forward	CTCGAGTCTTTTTAACTCGC
MACP2-1 Realtime qPCR	Reverse	CATGCTTCAGTACAAGACTTC
MACP2-2 Realtime qPCR	Forward	GCCTTGGGTTAACAATCCAAC
MACP2-2 Realtime qPCR	Reverse	GGCCTCTTACCAACATCAAC
MACP2-3 Realtime qPCR	Forward	TGATTTTGGTCTGGCCAACA
MACP2-3 Realtime qPCR	Reverse	TGGTTGAACTGCTCTGCCAT
MYB34-Realtime qPCR	Forward	TCACAAACCGATCAATTCAACC
MYB34-Realtime qPCR	Reverse	AAGCCGGAATATGTAGTCGTAG
MYB51-Realtime qPCR	Forward	ACACCAGTTTCATCGAACTTTG
MYB51-Realtime qPCR	Reverse	GTTTTCAACACAAGACTCCTCC
MYB122-Realtime qPCR	Forward	GACCAAAAGCTTATCGCCTATG
MYB122-Realtime qPCR	Reverse	AATCTTCCTCGTCTTGGCTAAA
MYB28-Realtime qPCR	Forward	AAAGAGGCGAGTTTAGTTCAGA
MYB28-Realtime qPCR	Reverse	AGTTCTTGATCTCGTTGTCTGT
MYB29-Realtime qPCR	Forward	TTCAAGCTCCAAAAATGTACCG
MYB29-Realtime qPCR	Reverse	GTAGAGCTGATCAAGGTTCCTT
MYB76-Realtime qPCR	Forward	CTGAAGAAAGGAGCATGGACTA
MYB76-Realtime qPCR	Reverse	TGGCAAATGTCTAGCTATGACA

Supplemental Table S1. Primers Used for Vector Construction and Quantitative Real-time qPCR Analysis
